# Periodontal Treatment Needs of Hemodialized Patients

**DOI:** 10.3390/healthcare9020139

**Published:** 2021-02-01

**Authors:** Agata Trzcionka, Henryk Twardawa, Katarzyna Mocny-Pachońska, Marta Tanasiewicz

**Affiliations:** Department of Conservative Dentistry with Endodontics, Faculty of Medical Sciences in Zabrze, Medical University of Silesia, Plac Akademicki 17, 41-902 Bytom, Poland; htwardawa@sum.edu.pl (H.T.); kpachonska@sum.edu.pl (K.M.-P.); martatanasiewicz@sum.edu.pl (M.T.)

**Keywords:** end-stage chronic kidney disease, hypertension, diabetes mellitus, periodontal status

## Abstract

End-stage renal failure is the reason for complications in many systems and organs, and the applied pharmacotherapy often causes the deepening of already existing pathologies within the oral cavity, such as: caries, periodontal diseases, mucosal lesions or reduced saliva secretion. Reduced saliva secretion results in an increased accumulation of dental plaque, its mineralization and prolonged retention, which leads to the development of gingival and periodontal inflammation. There is some evidence that chronic kidney diseases are influenced by periodontal health. The aim of the work was to evaluate the dental needs by the usage of clinical assessment of periodontal tissues of patients suffering from end-stage chronic kidney disease, arterial hypertension or/and diabetes mellitus. Material and methods: 228 patients underwent the research. 180 patients were hemodialized in Diaverum dialysis stations (42 of them were diagnosed with end stage chronic disease, 79 with the end stage chronic disease and arterial hypertension, 16 with end stage chronic kidney disease and diabetes, 43 with end-stage chronic disease, arterial hypertension and diabetes) and 48 patients of the Conservative Dentistry with Endodontics Clinic of Academic Centre of Dentistry of Silesian Medical University in Bytom and patients of the dentistry division of Arnika Clinic in Zabrze not diagnosed with any of the aforementioned diseases. The scheme of the research comprised 2 parts: analysis of the general health and assessment of the periodontal status which contain the following indices: Periodontal Probing Depth (PPD), Clinical Attachment Lost (CAL), Bleeding Index or Bleeding on Probing Index (BI or BOP), Community Periodontal Index for Treatment Needs (CPITN). Results: Significantly lower percentage of patients with healthy periodontal tissues and higher percentage with periodontal pockets deeper than 3.5 mm and the loss of trainers connective of 5 mm or higher were in the examined group. The values of the bleeding index were significantly lower in control group. The analysis of the CPITN index indicates higher percentage of patients qualified as CPI 1 or 2 in the control group while in the examined one most of the patients turned out to require specialist periodontal treatment. Conclusions: there is a direct relationship between periodontal status and end-stage renal disease which typically includes other chronical civilization ailments. It is important to develop a scheme for the easy and rapid examination of periodontal status, to determine the treatment needs in this area, which will allow precise assignment of long-term dialyzed patients to the range of prophylactic and therapeutic procedures.

## 1. Introduction

Chronic kidney disease (CKD) is now more common than ever. The increased incidence of kidney diseases allows us to classify them apart from cardiovascular diseases, hypertension, obesity and diabetes mellitus to the group of diseases of modern civilization of the 21st century [1,2]. In Poland the number of patients with CKD is estimated at 4 million, and in the whole world 600 million people [2]. The definition of CKD was established in 2002 by KDOQI (Kidney Disease Outcome Quality Initiative) and subsequently accepted by the international organization KDIGO (Kidney Disease Improving Global Outcome) [3]. This definition assumes that CKD is a multi-symptomatic disease syndrome resulting from permanent damage or reduction of active nephrons damaged by various disease processes in the kidney parenchyma. The CKD was divided into stages, depending on the degree of kidney function, and the definition of KDOQI was supplemented by a team of Polish nephrologists with the addition of nephrotic damage to reduce their number. The damage to nephrons must be permanent and the eGFR reduction (estimated glomerular filtration rate) should be understood as a decrease lasting at least 3 months [3]. The reasons for end-stage renal disease (ESRD) include diabetic nephropathy, chronic glomerulonephritis and hypertensive nephropathy. In the initial stage, ESRD can be asymptomatic, and the slow build-up of symptoms in the more advanced stages allows the patient to get used to them slowly and they may not experience any disturbing symptoms for a long time [1,2,4]. While the diagnosis and early detection of kidney diseases is relatively inexpensive, renal replacement treatment of one patient is approximately $50,000 and a kidney transplantation $17,000 [3]. Although there is a general consensus in Polish society for the most optimal renal replacement treatment, i.e., transplantation an organ from a deceased donor (supported by legislation and the position of the Catholic Church), the number of transplants is decreasing from year to year [1,2].

End-stage renal failure is the reason of complications from many system and organs, and the applied pharmacotherapy often causes the or deepening of already existing pathologies within the oral cavity, such as: caries, periodontal diseases, mucosal lesions or reduced saliva secretion. Reduced saliva secretion results in an increased accumulation of dental plaque, its mineralization and prolonged retention, which leads to the development of gingival and periodontal inflammation. Other factors predisposing to periodontal disease occur in ESRD patients are conducted with increased saliva pH caused by higher urea concentration and disorders of cellular and humoral immunity [5,6,7]. Hemodialized patients are predisposed to increased bleeding due to heparin administrated immediately before the procedure, which is necessary for the dialysis process [8,9]. Additionally, the mechanism of teeth surface cleaning is impaired by the deficiency of saliva buffer systems, which leads to a rapid increasing of caries. The mucosa symptoms such as atrophy, *Candida albicans* infections or erosions, combined with atrophy of the alveolar processes of the jaws, may cause difficult or even impossible use of removable dentures, which in turn hinders intake of food and, eventually, may lead to dietary impoverishment and malnutrition [6]. Significant is also the fact of manual limitation resulting from motor disability, which often characterizes older patients with ESRD. More than half of the dialyzed patients with their natural dentition visit the dentist less frequently then once every 5 years, and the vast majority of them go to the dentist’s office because of the sudden toothache [5,7]. Definitely better oral status is characterized by younger patients and those who are qualified for transplantation, whose failure to have their oral status improved before may complicate or even stop the procedure of transplantation [10].

On the other hand, increasing oral complaints in the form of untreated caries, advanced periodontal diseases, and even negligence of hygiene significantly affect the course and prognosis of systemic diseases. Oral microbiota homeostasis is important to maintain the state of oral health. Each dysbiosis or unbalanced spread of pathogens gives rise to several infectious diseases of both teeth and mucosae, which can perseverate in a chronic condition affected in status of the entire body [11]. There is a deep association between periodontitis and endocarditis which has been proven by several studies [12] or poor pregnancy outcomes [13]. There is some evidence that chronic kidney diseases are influenced by periodontal health [14,15]. In a prospective cohort of hemodialysis patients in Thailand, successful non-surgical periodontal treatment resulted in marked reductions in CRP after two months [15]. Limited information about the effect of periodontal therapy on patients receiving renal dialysis exists. Kshirsagar et al. demonstrated that treatment of periodontal disease improved CRP values in the dialysis population [16].

The aim of the work was to evaluate the dental needs by the usage of clinical assessment of periodontal tissues of patients suffering from end-stage renal disease, arterial hypertension or/and diabetes.

## 2. Materials and Methods

A total 228 patients took part in the study. The examined group (E) consisted of 180 patients treated in Diaverum dialysis stations in Katowice, Głubczyce, Warszawa and Kraków, who were diagnosed with the end-stage chronic kidney disease (ESRD). The patients were divided into 4 subgroups on the basis of the general disease they were diagnosed with (the information regarding the general health of the patients were obtained on the basics of the medical history obtained from the dialysis stations’ doctors):

R- end-stage chronic kidney disease (42 patients);

R + H- end-stage chronic kidney disease and hypertension (79 patients);

R + D- end-stage chronic kidney disease and diabetes mellitus (16 patients);

R + H + D- and end-stage chronic disease, hypertension and diabetes mellitus (43 patients).

The control group (C) was composed of patients from the Conservative Dentistry with Endodontics Clinic of the Academic Center of Dentistry in Bytom, the Medical University of Silesia in Katowice in addition to patients of the Dentistry Division of the Arnika Clinic in Zabrze, who had not been previously diagnosed with any of the civilization diseases included in the examined group. That group consisted of 48 people. Patients to both control and examined groups were chosen randomly [7].

### 2.1. Inclusion Criteria

The examined group consisted of patients aged > 40, suffering from diagnosed end-stage chronic kidney disease, blood hypertension and/or diabetes for at least 2 years, who had given their written consent to take part in the research.

The control group consisted of patients aged > 40, who were not diagnosed with any civilization disease included in the examined group, who had given their written consent to take part in the research.

### 2.2. Exclusion Criteria

Patients were excluded if they were aged under 40, had not given their written consent to take part in the research, pregnant women, with an acute phase of general diseases, incapacitated or diagnosed with assessed diseases for less than 2 years [7].

### 2.3. Methods

The research consisted of two parts. In first one, the general health of patients was analyzed (on the basic of the medical history, after consultation with the doctor at the dialysis station) and information regarding general diseases (especially hypertension and diabetes mellitus) was gathered.

In the second part the periodontal status assessment was undertaken and the following indices were measured:

- Periodontal Probing Depth (PPD) The measurement was recorded with the periodontal probe, which was introduced to sulcus or pocket with a little pressure (10–20 g). A probe was introduced parallel to root surface into the sulcus. The first incision seen above the sulcus was its size.
- Clinical Attachment Lost (CAL). This is defined as a distance between cementum enamel junction (CEJ) and gingival margin (GM). It was measured with the usage of periodontal probe. Received value was then added to the value of PPD: CAL = PPD + (CEJ + GM).
- Bleeding Index or Bleeding on Probing Index (BI or BOP). The assessment was undertaken with a dental probe. All four surfaces of each tooth were assessed. Providing that a patient had all teeth, maximally 128 surfaces (32 teeth × 4 surfaces) were assessed. In patients diagnosed with end- stage chronic disease the index was checked before heparin was given. In the case of small bleeding the dental floss was used. It was placed near the tooth surface in labial- lingual direction to the bottom of the sulcus. Then it was moved twice in apex to crown direction. In the next stage, floss was removed and the assessed region observed for 15 s (bleeding or no bleeding). If no bleeding was observed the noted value was 0, if there was bleeding 1. For each patient, the index was calculated by summing the values received for all teeth. Maximal index value was 128 and minimal 0. The lower the value, the better the sulcus condition.
- Community Periodontal Index for Treatment Needs (CPITN) by Ainamo, Barmes, Beadre et al. This index makes it possible to assess quickly and precisely the condition of periodontium, treatment needs and time for their realization. Teeth were divided into 6 sextants (17–14; 13–23, 24–27; 47–44; 43–33; 34–37). The examination was undertaken with a dental mirror, periodontal probe and dental probe. In each sextant all teeth were examined but only the highest value was noted.

The scoring was as follows:

0- healthy periodontium;
1- bleeding on probing, no periodontal pockets, no calculus nor overhanging restorations;
2- periodontal pockets to 3 mm, calculus or dental plaque above and below the gum level, overhanging restorations;
3- periodontal pockets 3.5–5.5 mm, bleeding on probing;
4- periodontal pockets ≥ 6 mm.

The criteria of treatment needs based on CPITN are presented in Table 1.

### 2.4. Statistical Analysis

The Kruskal–Wallis test was performed in order to assess differences in the averages of the parameters across the 5 groups. In the case of significant differences between the averages, the Mann–Whitney U test was performed to verify the accuracy of these differences. All these tests were performed using the IBM’s SPSS Statistics 23 program (IBM, Armonk, NY, USA), and *p* < 0.05 was considered indicative of a statistically significant result.

## 3. Results

The characteristics of the patients are presented in Table 2.

In order to obtain information regarding patients’ frequency of dental appointments, they were asked when their last appointment took place. Possible answers and results obtained are presented in Table 3.

The periodontal status was assessed by measurement of PPD, CAL, BI and CPITN. On the basis of the data received during the PPD and CAL assessment, patients were divided into 3 groups (Table 4).

No treatment needs and qualification to P1 group was observed in 45% of patients from C group, 6% of R group, 5% of R + H group. None of hemodialized patients with diabetes mellitus (R + D and R + H + D groups) was qualified to that group. A periodontist’s consultation and constant care of a dentist were needed in 45% patients from C group, 45%- R, 39%- R + H, 67%- R + D and 52%- R + H + D. The most advanced changes in periodontal tissues and need of specialist treatment were observed in 11% of patients from C group, 64%- R, 73%- R + H, 33%- R + D and 76%- R + H + D. The results of the *Chi-Quadrat* test for PPD and CAL are presented in Table 5.

**Figure 1:**
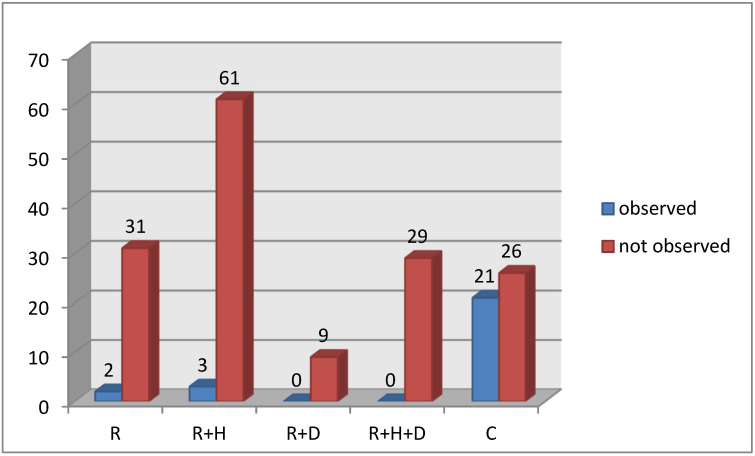
P1 changes in periodontium in groups.

In all subgroups of hemodialized patients (R, R + H, R + D, R + H + D) the percentage of patients with healthy periodontium was significantly lower than in control group C. In hemodialized patients the highest percentage of patients with healthy periodontium was in R and R + H groups (Figure 1).

**Figure 2:**
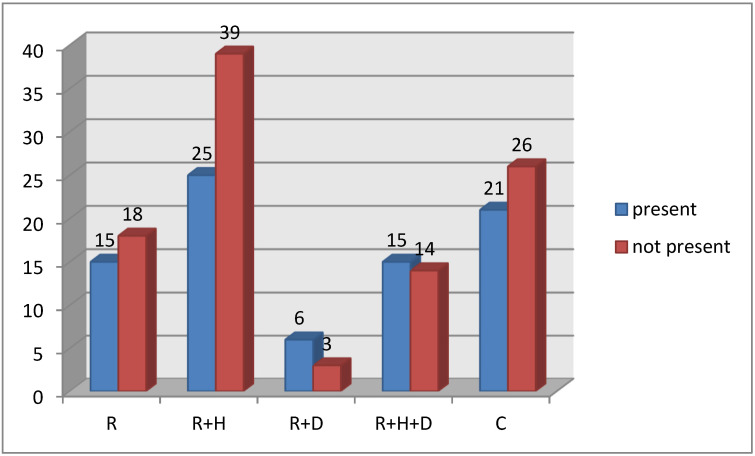
P2 changes in periodontium in groups.

There were no significant differences in the presence of P2 changes between groups (Figure 2).

**Figure 3:**
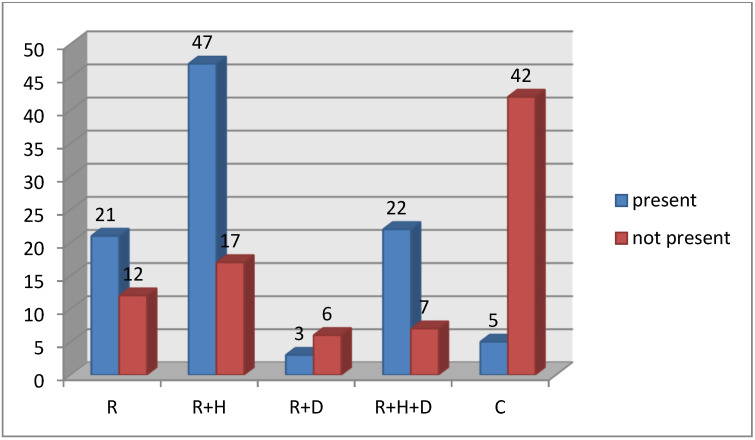
P3 changes in periodontium in groups.

In groups of hemodialized patients there was a significantly higher percentage of patients with P3 changes in comparison to healthy patients. In hemodialized patients the lowest percentage of patients with P3 changes were observed in patients with diabetes mellitus (Figure 3).

**Figure 4:**
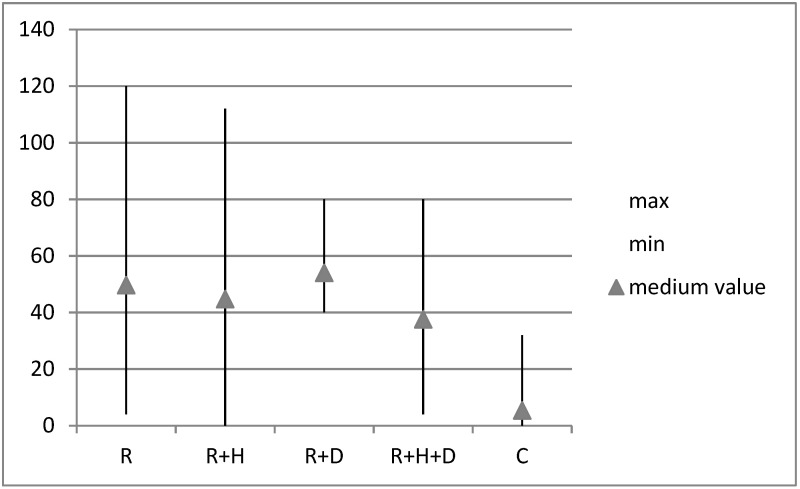
Minimal, maximal and average values of bleeding index in groups.

Bleeding index was not assessed in toothless patients. In control group (C) the minimal obtained value was 0 and maximal- 32. In particular subgroups of the examined group, the minimal and maximal values were as follows: R: 4, 120; R + H: 0, 112; R + D: 40, 80; R + H + D: 4, 80 (Figure 4).

**Figure 5:**
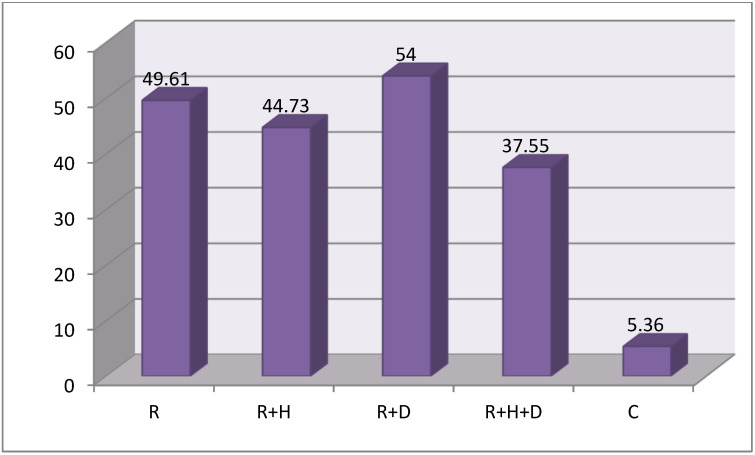
The average values of Bleeding Index (BI) in groups.

On the basics of Kruskal–Wallis test results it was confirmed that there were statistically significant differences between the groups BI χ^2^(4) = 89.83; *p* < 0.001; ε^2^ = 0.49. In order to check differences between the groups the U-Mann–Whitney test was undertaken. It proved that the average value of BI in control group (C) was significantly lower than in hemodialized patients (R, R + H, R + D, R + H + D) (*M* = 5.36; *SD* = 8.20 vs. *M* = 49.61; *SD* = 29.21 and *M* = 44.73; *SD* = 25.30 & *M* = 54.00; *SD* = 19.54 & *M* = 37.55; *SD* = 20.96) (Figure 5). Differences between the groups were moderate ε^2^ = 0.49.

### CPITN Index

The results obtained made it possible to divide patients into subgroups compatible with the CPI (0,1,2,3 or 4). Table 6 presents the number of patients classified as CPI-0, CPI-1, CPI-2, CPI-3 and CPI-4.

It needs to be said that only in 6 patients from examined groups (R-2, R + H-4) were there no pathological changes. In R + D and R + H + D there were no patients with a healthy periodontium while in control group (C) it was 13 people. Most of the patients from examined group were classified to the second category of treatment needs (TN) due to the CPITN index, while most of the patients from the control group to were classified to the first category (Table 7).

In patients from the control group (C) the percentage of patients with CPI 1 or CPI 2 was significantly higher and patients with CPI 3 or CPI 4 significantly lower than in all subgroups of the examined group.

A summary of the results is as follows:

- In examined group there was significantly lower percentage of patients with healthy periodontium and higher percentage of patients with periodontal pockets >3.5 mm and the periodontal attachment lost was 5 mm and more in comparison to the control group. While comparing particular subgroups in examined one it was observed that the highest percentage of patients with healthy periodontium was in R and R + H groups and the lowest with the highest number of people with advanced changes in the periodontium were in the R + D subgroup.
- The average values of bleeding index were significantly lower in patients belonging to control group than in the hemodialized group.
- In the control group a higher percentage of patients without or with moderate changes in periodontium (CPI 0, 1 and 2) was observed, while in the examined groups most of the patients had CPI 2 and higher, which classified them for specialist treatment.

## 4. Discussion

In patients with end-stage renal disease several changes in periodontal tissues may be observed, such as: lesions of alveolar bone, clinical attachment lost or periodontal pockets [9,10,17,18]. In own research, it was observed that in hemodialized patients there was a significantly lower percentage of patients with healthy periodontium (patients with sulcus of 0.5 mm depth, CAL ≤ 2 mm who do not have to be treated by a periodontist). Additionally, in hemodialized patients the highest percentage of people with healthy periodontium was in R and R + H groups. In the examined groups a significantly higher percentage of patients with PPD > 3.5 mm, CAL ≥ 5 mm who needed a periodontist’s treatment was observed. Comparison of examined groups showed that there was the lowest percentage of patients demanding specialist treatment in people diagnosed with diabetes mellitus. However, there was no patient with a healthy periodontium in patients with diabetes mellitus. The obtained results confirm the reports that in patients with diabetes mellitus the condition of the periodontium worsens. Similar results were obtained by Wilczyńska-Borawska et al., who proved, that the highest number of patients with healthy periodontium (CAL = 0) was in hemodialized patients without diabetes mellitus in comparison to those with diabetes mellitus [19]. The number of teeth with CAL ≥ 5 mm was the highest in patients with diabetes mellitus (especially in women) [19]. Clinical attachment lost was also assessed by Teratani et al. [20]. Despite the fact that they did not observe statistically significant differences in the percentage of teeth surfaces with CAL > 4 mm between three examined groups (hemodialized with or without diabetes mellitus, healthy patients), they noted that there was the highest percentage of teeth surfaces with CAL > 4 mm (28.3%) in patients with diabetes mellitus in comparison to hemodialized without diabetes mellitus 21.3% and healthy patients- 21.5% [20]. Jenebian et al. showed higher average values of CAL in hemodialized patients (4.39 ± 1.57 mm) in comparison to control group composed of healthy people (3.53 ± 1.56 mm); however those differences were not statistically significant. They observed significant differences comparing CAL values in hemodialized patients with regard to how long the hemodialysis lasted: the longer the time, the higher the values obtained [21]. Cchokra et al. observed statistically significant higher values of CAL in hemodialized patients (3.40 ± 0.68 mm) in comparison to healthy people 2.43 ± 0.33 mm) [22]. They observed a similar dependence for average values of pockets depth, which were as follows: in examined group- 2.82 ± 0.49 mm, in control- 2.12 ± 0.41 mm [22]. Jenebian et al. observed higher average values of periodontal pockets depth in hemodialized patients: 4.41 ± 1.53 mm than in healthy ones: 4.39 ± 1.23 mm [21]. Similarly to clinical attachment the differences were not statistically significant, however comparing those values in the hemodialized patients group they observed significant differences with regard to how long the hemodialysis lasted: the longer the period, the higher the values [21]. Swapna et al. observed moderate statistically significant difference in presence of periodontal pockets ≥6mm- they observed them in 23.4% of hemodialized patients with diabetes mellitus [23]. Vesterinen et al. observed periodontal pockets with depth higher than 5 mm in 45% of hemodialized patients with diabetes mellitus and in 34% of hemodialized patients without diabetes mellitus [24]. Teratani et al. showed that the frequency of occurrence of periodontal pockets with depth > 4 mm was significantly lower in patients diagnosed with end stage chronic kidney disease and diabetes mellitus (5.9%) than in healthy (9.9%) patients [20].

In order to assess the condition of periodontal tissue, the bleeding (BI) index was calculated. The average value of BI in control group was significantly lower than in examined groups. Teratani et al. obtained similar results [20]. Statistically significant, higher values, they obtained in hemodialized patients with diabetes mellitus in comparison to healthy patients [20]. Wilczyńska-Borawska et al., analyzed two bleeding indices, observed higher values of both in hemodialized men with diabetes mellitus and did not observe bleeding in any women with end-stage chronic kidney disease and diabetes mellitus [19].

Our results made it possible to assign patients to one of the subgroups due to the CPI index (0, 1, 2, 3 or 4). In patients from the control group the percentage of people classified to CPI 1 and 2 was significantly higher while the percentage of patients CPI 3 and 4 significantly lower than in all subgroups of the examined group. Only in 6 patients from the examined group were there no pathological changes in periodontium (from R and R + H groups). In groups R + D and R + H + D there were no people with a healthy periodontium, while in the control group we had 13 patients. The obtained results may suggest that the occurrence of general diseases such as end-stage chronic kidney disease and diabetes mellitus may influence on the treatment needs of periodontium. Most of the patients from that group was classified to the second category of treatment needs due to the CPITN index, while most of the healthy patients were classified to first category. Parkar et al. assessing the CPITN index observed that the highest percentage of hemodialized patients had changes in periodontium described as CPI 3 (51.97%) and CPI 2 (43.42%); in most cases of healthy patients they observed CPI 2 (77.63%) [25]. In both control and examined groups they did not observe people with a healthy periodontium (CPI 0). Only three patients from examined group (1.97%) obtained CPI 4 [25]. Swapna et al. compared CPI values between hemodialized patients with or without diabetes mellitus [23]. They observed moderate statistically significant higher frequency of CPI 4 occurrence in patients with diabetes mellitus (23.4% in comparison to 6% of patients without diabetes mellitus) [23]. The results obtained are of considerable importance for the treatment and stabilization the course of many chronic diseases, including those requiring continuous dialysis. There are reports in the literature that support the authors’ findings regarding the impact of chronic civilizational diseases on oral health [5,6,7,8]. In recent years the correlation between periodontal disease (resulting from biotom imbalance) on the course, prognosis and effectiveness of treatment of chronic diseases has also been emphasized [11,12,13,14,15]. There is evidence suggesting that there is a direct relationship between the inflammation of periodontal disease and chronic kidney diseases, and renal function may decline for various reasons. Chronic kidney disease evolves and/or coexist with diabetes, hypertension, and chronic nephritis [20]. The coexistence of multiple intractable conditions further exacerbates the patient’s oral situation, including an impact on their socio-economic status, thereby intensifying limitations in accessing necessary services [2,7].

## 5. Conclusions

There is a direct relationship between periodontal status and end-stage renal disease which typically includes other chronic civilizational ailments. Therefore, it seems important to develop a scheme for the easy and rapid examination of periodontal status in order to determine the treatment needs in this area, which will allow precise assignment of long-term dialyzed patients to the range of prophylactic and therapeutic procedures.

## Figures and Tables

**Table 1:** Treatment recommendations in relation to needs as determined by Community Periodontal Index for Treatment Needs (CPITN).

Clinical Assessment	Category of Treatment Needs According to CPITN
0 1	I. Oral hygiene instructions.
2 3	II. Oral hygiene instructions. Periodontal treatment consisted of removal of supra–and subgingival microbial deposits via scaling using hand and ultrasonic instruments. Correction of overhanging restorations.
4	III. Oral hygiene instructions. Periodontal treatment consisted of removal of supra–and subgingival microbial deposits via scaling and root planning (SRP) under local anesthesia using hand and ultrasonic instruments. Correction of overhanging restorations. Complex periodontal treatment.

**Table 2:** Patients’ characteristics.

Group	Sex		Avarage Age
	Men [*n*]	Women [*n*]	
C	15	33	52.71
R	25	17	67.21
R + H	48	31	62.54
R + D	10	6	70.19
R + H + D	27	16	72.86

**Table 3:** Answers to question about last dental appointment [7].

When Did You Last Visit a Dentist?		C	R	R + H	R + D	R + H + D
less than 3 months ago	number [*n*]	34	8	17	3	4
	% of group	71%	19%	22%	19%	9%
3–6 months ago	number [*n*]	5	6	13	2	9
	% of group	10%	14%	17%	13%	21%
a year ago	number [*n*]	7	13	17	7	11
	% of group	15%	31%	22%	44%	26%
I do not remember	number [*n*]	2	15	30	4	19
	% of group	4%	36%	39%	25%	36%

**Table 4:** Criteria of patients’ assignment to particular group dependent on periodontium status.

Group	Description
P1	patients with PPD ≤ 0.5 mm and CAL ≤ 2 mm, no periodontium treatment needed
P2	patients with PPD ≤ 3.5 mm and CAL 3–4 mm, periodontist consultation needed and constant control of a dentist
P3	patients with PPD > 3.5 mm and CAL ≥ 5 mm, specialist periodontium treatment needed

**Table 5:** The results the *Chi-Quadrat* test for PPD and CAL.

Variable	Result of *Chi-Quadrat* Test			
	X^2^	df	p	η
P1	48.43	4	0.000	0.52
P2	3.15	4	0.533	0.13
P3	53.64	4	0.000	0.54

**Table 6:** The analysis of the number of patients with regard to CPI index.

Group	Periodontal Symptoms CPI				
	Frequency (Percentage Distribution)				
	0	1	2	3	4
R	2 (6%)	7 (21%)	13 (39%)	3 (9%)	8 (24%)
R + H	4 (6%)	19 (30%)	19 (30%)	8 (13%)	14 (22%)
R + D	0 (0%)	1 (11%)	6 (67%)	0 (0%)	2 (22%)
R + H + D	0 (0%)	3 (10%)	12 (41%)	6 (21%)	8 (28%)
C	13 (28%)	14 (30%)	10 (21%)	6 (13%)	4 (9%)

**Table 7:** The analysis of the number of patients with regard to treatment needs (TN).

Group	Treatment Needs TN		
	Frequency (Percentage Distribution)		
	I	II	III
R	9 (28%)	16 (48%)	8 (24%)
R + H	23 (35%)	27 (42%)	14 (22%)
R + D	1 (11%)	6 (67%)	2 (22%)
R + H + D	3 (10%)	18 (62%)	8 (28%)
C	27 (57%)	16 (34%)	4 (9%)

## Data Availability

The data presented in this study are available on request from the corresponding author.
